# Effect of Thermal and Non-Thermal Processing on Nutritional, Functional, Safety Characteristics and Sensory Quality of White Cabbage Powder

**DOI:** 10.3390/foods11233802

**Published:** 2022-11-25

**Authors:** Muhammad Waseem, Saeed Akhtar, Muhammad Qamar, Wisha Saeed, Tariq Ismail, Tuba Esatbeyoglu

**Affiliations:** 1 Department of Food Science and Technology, Faculty of Agriculture & Environment, Islamia University, Bahawalpur 63100, Pakistan; 2 Faculty of Food Science and Nutrition, Department of Food Science and Technology, Bahauddin Zakariya University, Multan 60800, Pakistan; 3 Institute of Food Science and Human Nutrition, Department of Food Development and Food Quality, Gottfried Wilhelm Leibniz University Hannover, Am Kleinen Felde 30, 30167 Hannover, Germany

**Keywords:** antinutrients, bakery, fiber, *Brassica oleracea*, fortification, functional food, leafy vegetables, microwave, mineral, pesticide

## Abstract

This study was aimed to improve nutritional, functional and consumer safety aspects of cabbage powder (CP). White cabbage (*Brassica oleracea* var. *capitata f. alba*) was dehydrated to CP following microwave heating, blanching, alkali or acid washing treatments. The results for nutrients and mineral composition of raw and processed CP elucidated raw CP to exhibit significantly (*p* < 0.05) higher amounts of protein (12.2%), dietary fiber (25.2%), Na (52 mg/100 g), Ca (355 mg/100 g), K (286 mg/100 g), Fe (14 mg/100 g) and Zn (32 mg/100 g). Among different processing techniques, microwave treatment resulted in a higher rate of reduction for alkaloids, oxalates, tannins and phytates contents, i.e., 77%, 85%, 85%, and 86%, respectively. Likewise, microwave treatment was found more effective in reducing residual levels of neonicotinoids, pyrethroids, organophosphates including imidacloprid, cypermethrin, bifenthrin, chlorpyrifos and deltamethrin in cabbage in the range of 0.98–0.12 ppm, 1.22–0.23 ppm, 1.03–0.15 ppm, 1.97–0.43 ppm, and 2.12–0.36 ppm, respectively. CP supplementation at the rate of 5% in unleavened flatbreads was observed to maintain textural and sensory attributes of the product. The results suggest microwave heating as a cost-effective technique to reduce toxicants load in cabbage powder. Further, ~5% supplementation of CP in wheat flour may also improve nutritional and functional properties of the baked goods.

## 1. Introduction

White cabbage (*Brassica oleracea* L. var. *capitata f. alba*) is a commonly consumed cruciferous green leafy vegetable that belongs to the Brassicaceae family [1]. Data on nutritional characterization of raw and processed cabbage suggest substantial load of dietary fibers, minerals, vitamins, and phytonutrients such as glucosinolates, polyphenols and flavonoids bearing anti-genotoxic, antioxidant and cytotoxic properties [2]. Therapeutic properties of cruciferous vegetables including cabbage against various health disorders are predominately linked to glucosinolate hydrolysis products, i.e., isothiocyanates. Additionally, bioactive polyphenols, vitamins and fibers of cabbage have also been documented to anticipate antioxidant, anti-inflammatory, anticancer and cardioprotective properties [1,3]. White cabbage holds a significant position among functional foods due to its extensive and beyond-food application in contemporary nutrition and traditional medicine. Modes of dietary uses of white, green, purple and red cabbage have been reported as fresh cut, fermented products such as sauerkraut and as dehydrated powders for salads [4] and other food preparations such as biscuits [5], soups, functional juices and weight-loss diets [6].

Anti-nutrients of biological and human origin are known to impair mineral bioavailability and adversely affects human health. Likewise, the indiscriminate application of health hazardous/poisonous pesticides on vegetable crops to kill insects and pests have been commonly observed in developing countries. In close nexus, the dietary intake of these pesticide contaminated vegetables results in significant harm to human health. Pesticide residues in vegetables are known to cause acute toxicity such as diarrhea, salivation, diaphoresis, excitation of nervous system and acute renal failure, while the effect of chronic dietary exposure had been reported with the risks of miscarriage, birth defects, neurological, behavioral and immunological disorders, memory loss, hypersensitivities and various forms of cancers [7]. The maximum residue limit (MRL) for various pesticides including has been listed in a range of 0.1–1.0 mg/kg for head cabbage [8,9]. A report from Wanwimolruk et al. [10] reveals the presence of organophosphates and carbamates such as carbofuran, dimethoate, fenobucarb and isoprocarb beyond the MRL in more than 80% samples of cabbage and kale.

A great interest has emerged among the research community in recent times to reduce the biological and environmental burden of the food toxicants to prevent ever increasing burden of chronic diseases [11]. Various non-thermal processing techniques including fermentation, alkali and acid soaking, and thermal processing have been reported to reduce the toxicants burden in fresh plant produce. Investigational studies on impact of invasive and non-invasive vegetables processing techniques commensurate reduction in magnitudes of the biochemical toxins may be attributed to their extent of solubility in water, heat susceptibility, extent, and the strength of ionic bonding among food components and the toxicants [11,12]. Microwave heating of vegetables had been reported to generate differential patterns of changes in antinutrients and phytochemical matrix, which may help in designing healthful and nutritionally promising cooking techniques [13].

Plausible information exists on physicochemical and anti-nutrient studies of dried cabbage and its food application. Contrarily, the literature identifies a gap in availability of a comparative study on various detoxification techniques for white cabbage and their impact on functional and nutritional properties of the processed good. Therefore, this research was designed to assess the effect of various thermal and non-thermal processing techniques to detoxify raw cabbage from a range of intrinsic and extrinsic toxicants, and to define maximum admissible supplementation level of the processed and dehydrated cabbage that has the least impact on sensory characteristics of the baked unleavened flatbread.

## 2. Materials and Methods

### 2.1. Raw Material, Chemical and Reagents

Fresh cabbage (*Brassica oleracea*) leaves (25 kg) were procured from Dua Enterprises-Agro-farm (Multan, Punjab, Pakistan). High-performance liquid chromatography (HPLC) grade, analytical grade chemicals and reagents were purchased from Sigma Chemical Co., Ltd. (St. Louise, MO) and Merck (Darmstadt, Germany) through local distributors. Standards for minerals (Na, Ca, K, Fe, Zn), anti-nutrients (alkaloids, oxalates, tannins, phytates) and pesticide residues (imidacloprid, cypermethrin, bifenthrin, chlorpyriphos, deltamethrin) were obtained from BDH Chemicals Ltd. (Shanghai, China).

### 2.2. Detoxification of Fresh Cabbage Leaves

Microwave Heating: Homogenously chopped fresh white cabbage leaves (2.0 kg) were placed in microwave for processing at 1.1 kW for 2 min.

Blanching: Uniformly shredded cabbage leaves (2.0 kg) were dipped in hot distilled water (1:10, *w*/*v*) at 100 ± 2 °C between 1–1.5 min and drained using perforated stainless steel screens and pre-dried with absorbent paper.

Acid and alkali soaking: The protocol of Amin et al. [14] was followed for acid and alkali soaking of cabbage. Freshly procured uniform pieces of white cabbage leaves (2.0 kg each) were dipped in 2 M hydrochloric acid (HCl) solution and 1% sodium bicarbonate and mixture of salt solutions (i.e., 0.5% and 1.5% of sodium carbonate and sodium bicarbonate solutions) for 16–18 h, respectively.

### 2.3. Cabbage Powder Preparation

Cabbage leaves before processing were washed with demineralized water to remove any adherent foreign particles and shredded with stainless steel knives. Shredded leaves of raw and processed white cabbage were evenly spread over the 0.186 × 0.186 m^2^ nylon mesh for cabinet dehydration (Pamico Tech, Faisalabad, Pakistan) at 50 ± 2 °C for a period of 10–12 h to reduce residual moisture contents of the cabbage up to 8–10%. Raw and processed dehydrated cabbage leaves were powdered ~80 mm mesh size in heavy duty grinder (Pamico Tech, Faisalabad, Pakistan) and stored in airtight glass containers at 4–6 ± 2 °C for further experiments.

### 2.4. Functional Properties of Raw and Processed Cabbage Powders

#### 2.4.1. Bulk Density (BD) and Rehydration Ratio (RR)

BD of the raw and processed cabbage powders was measured by following the method of Shafi et al. [15]. RR of raw and processed CP samples was estimated in accordance to Yu et al. [16] with slight modifications. Accurately measured 5 g of each sample was directly poured into 50 mL clean distilled water and was persisted for 30 min, at room temperature. Afterwards, mixture was filtered (Whatman no. 41), and the permeate of each sample was accurately weighed. The rehydration ability of each sample was measured using the following formula;

(1) $$\left(\mathrm{RR}\right)\;=\;\frac{\mathrm{Weight}\;\mathrm{of}\;\mathrm{drained}\;\mathrm{material}\;\left(W1\right)\;\left(g\right)}{\mathrm{Weight}\;\mathrm{of}\;\mathrm{dehydrated}\;\mathrm{residues}\;\left(W2\right)\;\left(g\right)}$$

#### 2.4.2. Water Absorption Index (WAI)

WAI was estimated using the method as adopted by Sharma et al. [17]. Briefly, 12.5 g each of the raw and processed cabbage powder samples were weighed and poured into the conical flask containing 15 mL distilled water. The mixture was subjected to stirring for 30 min, and contents were centrifuged (Hermle Z236K, Wehingen, Germany) at 4300 rpm for 20 min. Supernatant were collected and dried, at 105 ± 2 °C, for 24 h in a drying oven (Memmert UNB 200, Schwabach, Germany). Results were expressed as the amount of water absorbed (%) per gram of the sample.

#### 2.4.3. Water Solubility Index (WSI)

AACC method No. 44-19 was used for measuring WSI of raw and processed cabbage powders [18]. One gram of sample was mixed in distilled water and heated in a water bath (WNB-29, Memmert, Schwabach, Germany), at 80 ± 5 °C, for 30 min. The mixtures were centrifuged at 4300 rpm for 15 min (Hermle Z236K, Wehingen, Germany) and were oven-dried (Memmert UNB 200, Munich, Germany), at 70 ± 2 °C, for 8 h. Water solubility index was calculated by the formula given below:

(2) $$\mathrm{WSI}\;\left(\%\right)\;=\;\frac{W1\;\left(g\right)\;-\;W2\;\left(g\right)}{W\;\left(g\right)}\;\times \;100$$

where *W* = weight of dehydrated sample, *W_1_* = weight of oven dried sample with Petri dish, and *W_2_* = weight of Petri dish.

#### 2.4.4. Swelling Power (Sp)

Swelling power of the raw and processed cabbage powders samples were determined in accordance with the method followed by Gani et al. [19]. Briefly, 3 g powder sample was mixed with 30 mL distilled water in 50 mL falcon tubes. The tubes were heated in submerged condition, at 70 ± 2 °C, for 30 min in a water bath (WNB-29, Memmert, Schwabach, Germany). Heated samples mixtures were centrifuged (Hermle Z236K, Wehingen, Germany) at 1500 rpm for 10 min. Supernatants were drawn off and while solid contents deposited in the tubes were weighed. Swelling power of the samples was calculated using the following formula:

(3) $$\left(\mathrm{Sp}\right)\;=\;\frac{\mathrm{Weight}\;\mathrm{of}\;\mathrm{solid}\;\mathrm{paste}\;\left(g\right)}{\mathrm{Weight}\;\mathrm{of}\;\mathrm{original}\;\mathrm{dry}\;\mathrm{powder}\;\mathrm{sample}\;\left(g\right)}$$

#### 2.4.5. Hygroscopicity (Hg)

Hygroscopicity of raw and processed variants of cabbage powder samples were estimated in accordance with previously described method [20]. Ten-gram sample was placed in desiccator, at room temperature (25 ± 2 °C), accompanied with sodium sulphate (Na_2_SO_4_) maintaining 80% relative humidity for 1 week and weighed afterwards. Hygroscopicity was measured as moisture absorbed by the sample in grams per 100 g of dry solids (g/100 g). Results were calculated using the following formula;

(4) $$\mathrm{Hygroscopicity}\;(\%)\;=\;\frac{∆m}{m\;+\;m1}\;÷\;1\;+\;\left(\frac{∆m}{m}\right)$$

where ∆*m* represents increase in powder’s weight at equilibrium (g), and *m* is the initial weight of powder (g), while *m_1_* is moisture free powder (g/100 g).

### 2.5. Proximate Composition

Proximate composition, i.e., moisture contents (Method No. 934-01), crude ash (Method No. 942-05), crude fat (Method No. 920-39), crude protein (Method No. 984-13), crude fiber (Method No. 978-10), and nitrogen-free extract of each raw and processed cabbage powder samples were determined in accordance with the AOAC methods [21].

### 2.6. Mineral Profiling

Wet digestion was performed to determine macro and micro minerals by atomic absorption spectrometry. Briefly, 0.5 g of the CP was mixed with shifted-to-Teflon vessels containing 5 mL of nitric acid and 2 mL of hydrogen peroxide. Acid digestion was performed for 1 h, and the digested samples were filtered and transferred to 50 mL volumetric flask. Volume of the flask was made up to 50 mL with deionized water. Micro and macro minerals were estimated on atomic absorption spectrometer (iCE 3000, Thermo Fisher, USA) using air–acetylene flame [19].

### 2.7. Determination of Anti-Nutritional Contents in Raw and Processed Cabbage Powders

#### 2.7.1. Estimation of Alkaloid Contents

Alkaloid contents in raw and processed samples of the cabbage powder were determined according to previously described method [22]. Briefly, five grams of the powdered samples was thoroughly mixed in 50 mL, 10% ethanol:acetic acid solution and allowed to react for 4 h. The reaction mixture was filtered through Whatman filter paper no. 41. Titration was performed with concentrated ammonium hydroxide to precipitate alkaloids. Resultant alkaloids’ precipitates were washed with 1% solution of ammonium hydroxide. Final filtration was achieved on pre-weighed filter paper. Alkaloid precipitates on filter paper were oven-dried, at 60 °C, for 30 min. Filter paper containing alkaloids’ precipitates were weighed accurately. Alkaloids (%) were estimated using the following formulae:

(5) $$\mathrm{Alkaloids}\;\left(\%\right)\;=\;\frac{\mathrm{WFPP}\;-\;\mathrm{WEFP}}{\mathrm{Ws}}\;\times \;\frac{100}{1}$$

Ws = sample weight (g), W_EFP_ = empty filter paper (g), W_FPP_ = weight of filter paper and precipitate (g).

#### 2.7.2. Determination of Oxalate Contents

Oxalate contents in raw and processed cabbage powder samples were estimated in accordance with the protocol followed by Amin et al. [14]. One gram of each sample was thoroughly mixed with 6 M, 10 mL HCl in a 250 mL conical flask. Final volume was adjusted to 190 mL using distilled water, and flask contents were left to stand for 1 h. Post-digestion, the mixture was centrifuged at 2000 rpm for 10 min. Thereafter, 50 mL of the centrifuged supernatant was concentrated up to 25 mL using hot plate (LMS-1003, Daihan Labtech Co. Ltd., Namyangju, South Korea) and filtered to collect brown precipitates. These precipitates were washed with deionized water and conc. ammonia solution to obtain a faint yellow or pink-colored precipitate. Furthermore, 10 mL (5%) of calcium chloride solution was added in it to obtain oxalates precipitates which were filtered and titrated against 0.05 M, KMnO_4_ to observe pink colored end-point persisting for 30 s. Oxalates were estimated by following formula:

*Oxalates* (*%*) = *Titer value* × 0.1125 (6)

#### 2.7.3. Determination of Tannin Contents

Folin–Denis reagent method was used to determine tannins in raw and processed cabbage powder samples [14]. One gram of each sample was heated in 75 mL distilled water (*w*/*v*) for 30 min and cooled. One milliliter of the supernatant was mixed with 0.5 mL Folin Denis reagent and 1 mL sodium carbonate (Na_2_CO_3_) (7.5%). Final volume of the mixture was adjusted to 10 mL using the distilled water, and reaction was completed by allowing 30 min incubation time. Absorbance of each sample was measured at 700 nm on UV-Vis spectrophotometer (UV-Vis 3000, Darmstadt, Germany). Tannin contents were recorded in mg/100 g against the standard curve of tannic acid.

#### 2.7.4. Determination of Phytate Contents

Haug and Lantzsch [23] method was adopted to determine the phytates in raw and processed cabbage powder samples. One gram of the powder sample was extracted for phytates with 0.2 N, 10 mL of HCl for 30 min on a hot plate (LMS-1003, Daihan Labtech Co. Ltd., Namyangju, South Korea). Afterwards, 0.5 mL of the extracted material was shifted into stoppered test tubes already containing 1 mL of ferric solution (i.e., 0.2 g ammonium iron III sulphate in 2 N, 100 mL HCl finishing to total volume of 1000 mL) and boiled in a water bath (Memmert WNB-29, Schwabach, Germany) for 30 min. Test tubes were cooled in ice water to room temperature (25 °C). Subsequently, centrifugation (Hermle, Z236K) was performed at 3000 rpm for 30 min. Thereafter, one milliliter of the supernatant was added in 1.5 mL of 2,2′-bipyridine solution (i.e., 0.25 g 2,2′-bipyridine and 0.25 g thioglycollic acid in distilled water up to final volume of 25 mL). Standards of phytate-phosphorous were prepared for the standard curves. Absorbance of reagent blank, standards and samples were recorded at 519 nm in UV-Vis spectrophotometer (UV-Vis 3000). Phytate contents were calculated against phytic acid standard curves using distilled water as reagent blank.

#### 2.7.5. Extraction, Clean-Up and Estimation of Pesticides Residues

Pesticide residues in raw and processed cabbage powder were extracted with ethyl acetate solvent. Briefly, 50 g of the homogenized sample and 20 g of anhydrous sodium sulfate were added in 250 mL conical flask and thoroughly mixed to prevent clod formation. After mixing, 75 mL ethyl acetate and 10 mL saturated sodium chloride solution were added with glass beads to improve extraction of the pesticide’s residues in an orbital shaker (MaxQ4000, Thermo Scientific TM, Waltham, MA, USA) at 240 rpm for 1 h. The extract was filtered using Whatman filter (No. 4). The filtered extract was placed in clean plastic bottles and stored at refrigeration (2–4 ± 2 °C) for further analysis.

Cleanup in column chromatography was performed to remove impurities in samples using the protocols as documented by Randhawa et al. [24]. A thin layer of anhydrous sodium sulfate was prepared on glass wool at bottom of the column. Subsequently, another layer of mixture of activated charcoal and silica gel 7:5 (*w*/*w*) was made. The prepared column cleanup system was adjusted at 1 mL per minute flow rate. Afterwards, the samples were eluted for clean-up process with 50 mL mixture of methanol and acetone (7:3 *v*/*v*) and eluted in 150 mL plastic bottles. The collected elute was concentrated to 1–1.5 mL in rotary evaporator at 40 °C. The cleaned-up elute concentrate was collected in glass vials using the glass sucker. Cleaned-up elute was washed with methanol. Nitrogen gas fluxing was performed under controlled pressure in Petri plates to remove impurities.

The residual contents of the extracted pesticides, i.e., imidacloprid, cypermethrin, bifenthrin, chlorpyrifos, and deltamethrin in raw and processed cabbage powder samples were assessed on an HPLC system equipped with a UV–visible detector (PerkinElmer, Series 200, Waltham, MS, USA). Accurately measured 20 µL of each sample was injected on a C18 column (Supelco, Bellifonte, PA, USA; 250 mm × 4.6 mm i.d.) using a gradient system installed with an autosampler at 30 °C. The mobile phase (methanol: water 45:55 *v*/*v*) was pre-set at a flow rate of 1 mL per minute. Sample processing run time for each sample was set at 20 min and imidacloprid, cypermethrin, bifenthrin, chlorpyrifos, and deltamethrin contents were detected at 254 nm. Samples were measured against the standard curves which were developed at concentrations of 2, 4, 6, 8, and 10 mg/kg accompanied with blank [24].

### 2.8. Sensory Assessment

Accurately measured 50.0 g dough was developed by mixing in optimum quantity of water and whole wheat flour which thereby converted into chapatis and baked at 200 ± 5 °C for 1–2 min on both sides. Sensory acceptability of the control (T_0_) (i.e., 100% wheat flour) and microwave heat-treated cabbage powder supplemented flatbreads at 2.5, 5, 7.5 and 10% supplementation levels were determined by 20 sensory panelists with better sensory discrimination capability from Institute of Food Science and Nutrition, Bahauddin Zakariya University, Multan. Panelists were asked respond against sensory parameters including appearance, color, folding ability, taste, texture and overall acceptability using 9-point hedonic scale.

### 2.9. Statistical Analysis

Each analysis was replicated twice as two independent experiments. The results of physical properties of the powder, biochemical composition and intrinsic as well as extrinsic toxicants were computed as means ± S.E. The results on sensory analysis of cabbage powder supplemented flatbread were computed as means ± S.D. Data obtained from raw and processed cabbage powder samples’ nutritional composition, physiochemical properties and sensorial study of processed cabbage powder-based flatbreads were statistically evaluated using ANOVA technique on Statistix 8.1 (Tallahassee, FL, USA) software. Level of significance of the means was estimated by using LSD test at 5% confidence level.

## 3. Results

### 3.1. Functional Characteristics of Raw and Processed CP

Data obtained from functional characteristics of the tested raw and processed CP samples presented significantly (*p* < 0.05) higher WSI and ^RR^ in raw cabbage powder itself, i.e., 4.8 and 5.1%, respectively, in comparison with different processed forms of powdered cabbage (Table 1). Comparison of treatment efficiency for various functional properties identified microwave processing, blanching and microwave processing to exhibit highest WAI, Sp and Hg, i.e., 6.91 g/g, 8.21 g/g and 7.60%, respectively.

### 3.2. Nutritional Composition of Raw and Processed Cabbage Powders

Composition of CP dried at ~10% moisture contents elucidated raw CP to hold promising nutritional composition that include ash (9.50%), crude protein (12.2%), crude fiber contents (25.2%), carbohydrates (40.8%) and crude fat (1.70%) (Table 2). With a preview of nutrient dense profile of cabbage powder, various processing techniques deployed in this study significantly reduced amounts of crude proteins, crude fat, and crude dietary fibers in processed powders. Among the processed treatments, acid soaking yielded highest amounts of ash (10.3%), blanching yielded highest crude protein (12.1%) and microwave heating anticipated higher crude dietary fibers contents (24.1%). Alkali-soaked cabbage leaves derived powder was carrying highest carbohydrates contents, i.e., 47.2%.

### 3.3. Mineral Composition of Raw and Processed Cabbage Powders

Leafy vegetables are modest source of micronutrients and hence play a pivotal role in maintaining better health conditions, and alleviating risks of micronutrient deficiencies. The comparative analysis of mineral composition of raw and processed cabbage powders indicated raw CP elucidates significantly higher mean concentration of Na, Ca, K, Fe, and Zn, i.e., 52, 355, 286, 14.0 and 32 mg/100 g, respectively, when compared to processed cabbage powders (Table 3). Significant losses in Zn, Fe and Ca contents were identified in CP developed form alkali-treated, acid-treated and blanched white cabbage leaves, respectively (Table 3). Comparison of mean minerals contents of various treatments exhibited non-significant (*p* > 0.05) differences suggesting matchable response of processing treatments on minerals profile of the CP excepting acid treatment of the white cabbage that led to a significant change in Fe and K contents of the processed good.

### 3.4. Reduction in Anti-Nutrients in Raw and Processed Cabbage Powders

The results of the study suggest raw CP to hold higher concentrations of alkaloids, oxalates, tannins and phytates, i.e., 184, 48.9, 1175 and 86.2 mg/100 g, respectively. While various processing treatments deployed in this study anticipated a significant (*p* < 0.05) decline in mean concentration of all anti-nutrients (Table 4). Among the treatments, microwave processing of raw cabbage leaves resulted in highest rate of reduction in alkaloids, oxalates, tannins and phytates, i.e., 77%, 85%, 85% and 86%, respectively, when compared with the anti-nutrients load of raw and other processing treatments.

### 3.5. Reduction in Pesticide Residues in Raw and Processed Cabbage Powders

Statistical findings on pesticide residues in raw and processed CP are presented in Table 5. The results suggest higher concentrations of imidacloprid, cypermethrin, bifenthrin, chlorpyrifos and deltamethrin in raw CP, i.e., 0.98, 1.22, 1.03, 1.97 and 2.12 ppm, respectively. Impact assessment of various processing techniques presented a significant effect (*p* ≤ 0.05) on pesticide residues of the CP when compared with the control and treatments (Table 5). Highest rate of reduction in imidacloprid, cypermethrin, bifenthrin, chlorpyrifos and deltamethrin residues were recorded in microwave-processed CP, i.e., 87, 80, 84, 77 and 83%, respectively, followed by blanching, acid soaking and alkali soaking.

### 3.6. Effect of Processed (Microwave-Treated) Cabbage Powder Substitution on Sensory Quality of Flatbreadss

Value addition of the baked goods with novel ingredients is usually expected to significantly influence sensory attributes and consumer acceptability of the finished products. Microwave-treated CP, on account of its better response to anti-nutrients’ reduction, was evaluated as potential natural wheat flour fortificant for developing unleavened flatbread. Data from the sensory panelists findings on various sensory traits of control (T_0_) and CP supplemented unleavened flatbread are given in Table 6. Hedonic rating of various sensory attributes of supplemented unleavened flatbread ranged between 7.6 and 6.1 (like moderately to like slightly) for all sensory traits of unleavened flatbread at 2.5–7.5% level of processed cabbage powder supplementation. Comparatively, poor scoring was assigned to all sensory attributes of flatbread carrying CP beyond the 7.5% level of supplementation. In comparison with the mean score of ~8.0 for 100% straight grade flour flatbread, the highest overall acceptability with mean score of ~7.6 was determined for flatbread prepared with 5% processed cabbage powder supplemented wheat flour.

## 4. Discussion

High WAI increases degree of solubilization of the starches. This may be associated with presence of more hydrophilic constituents such as polysaccharides and proteins in cabbage powder, as reported by Chandra et al. [25]. Relatively higher WAI and Sp in CP may be associated with comparatively higher protein contents and their interaction with water molecules. High WAC and degree of solubilization are intended to increase viscosity, as has been desired in baked goods, soups and gravies. Unlike CP, lower WAI has been recorded for non-wheat flours including cowpea flour (2.3 g/g) and maize flour (1.7 g/g) [25], suggesting cabbage flour as a potential candidate for composite flour in bread making. Sp of cabbage powder was observed higher than wheat flour. Relatively higher values of Sp have been reported for cassava (13.8 g/g) and cowpea (12.1 g/g) which may be associated with comparatively higher concentration of amylopectin than observed in CP and wheat flour [26]. Compared with cassava and cowpea, head cabbage is widely cultivated and consumed in Pakistan, and thus, it tends to be a promising and economical alternate to conventional sources of amylopectin. Better functional properties, specifically, WAI, Sp and Hg, were reflected in microwave-processed CP as compared to the raw, and similarly to the powder developed from blanched, alkali and acid soaked cabbage leaves.

Nutritional facts of dehydrated cabbage agreed to the findings of a previous study, wherein ash, protein and dietary fibers were reported as 7.8%, 11.4% and 61.4%, respectively [27]. The reduction in nutrients levels of the processed CP might be associated with the thermal degradation of the nutrients, solubility of hydrolysable nutrients and their leaching losses. Reduction in protein contents of the CP may be associated with the physico-chemical changes such as unfolding of protein structures, breakdown of intra-linkages and denaturation. Protein contents of the CP are comparable to the local wheat cultivars, thus suggesting partial replacement of the wheat flour with dehydrated cabbage to not significantly affect protein-energy contents of the blend. However, CP supplementation in low protein carrier staple cereals and the tuber flours may significantly enhance food value of the blends. In South East Asia, where wheat is a primary staple cereal, partial substitution of wheat flour by CP fortification may further help in complimenting essential amino acids including lysine, leucine, threonine, phenylalanine and valine.

Earlier research by Warman and Havard [28] on mineral composition of organic and conventional dehydrated cabbage had reported relatively higher levels of Ca (312–614 mg/100 g) and Na (67–93 mg/100 g) than observed in our study. The findings of the current study are in close agreement with the report from Umoh and Iwe [29] on tuber vegetable crop, stating a significant (*p* < 0.05) decline in mineral elements in blanched and sun-dried false yam tuber flour. The statistical findings revealed decline in Na (19–11 ppm), Ca (98–12 ppm), K (32–27 ppm), Fe (3–1 ppm), and Zn (2–1 ppm) contents, respectively. Green leafy vegetables are reported as highly susceptible foods for microelements losses during various cooking operations. In another study [30], the authors suggested mass cooking techniques to anticipate 30–40% losses in minerals contents of the cooked foods particularly vegetables. Possible causes of the microelements losses in vegetables may include outflow of the minerals from the food matrix treated either with direct or indirect heat, acids or alkali [30].

Inadequate dietary intake of plant centric foods is one amongst the leading causes of health and nutritional calamities both in industrialized and impoverished populations of the developing world. It is evident from the present study that both the raw and processed variants of CP hold plenty of essential elements, particularly Fe and Zn, which might anticipate an essential role in alleviating micronutrients malnutrition among vulnerable populations. Though the results on effect of various processing techniques on minerals contents of the CP are largely non-significant, yet microwave dry-heating of cabbage leaves, unlike water blanching and soaking, has less susceptibility to leaching effects on soluble fractions of the minerals more likely the divalent cations.

Anti-nutrients such as oxalates, tannins and phytates are generally recognized as toxicogenic compounds which chelate divalent cations such as Ca, Mg, Fe, Zn and Cu. Lead binding of essential elements by toxins reduces minerals bioavailability for humans and leads to risks of micronutrient malnutrition. Anti-nutrients such as phytates, tannins, alkaloids, oxalates and cyanogenic glycosides are evidently reported to adversely affect nutrients bioavailability by affecting starch and protein digestion and absorption, and chelating divalent cations including Fe, Zn and Ca. Thermal degradability of the listed antinutrients and susceptibility of acids toward hydrolyzing nutrients-toxicants bonding has been reported to markedly improve nutrients bioavailability for their predefined metabolic role. Microwave heat pre-treatment, which has been found more promising than the counterpart processing techniques, has a quite different mechanism of action than soaked blanching, cooking. Thermal instability of antinutrients, their susceptibility to leaching and hydrolysis of peptides bonds are amongst the key antinutrient reducing mechanism for soaked thermal treatments. Degradation of the antinutrients by microwave cooking, however, involves thermal damage alongside formation of insoluble complexes between the antinutrient and non-nutrient compounds, generation of free radicals, and destruction of sulfhydryl and the disulfide groups [31,32,33].

In a study by Mosha et al. [31], cabbage processing by blanching elucidated significant (*p* < 0.05) reduction in phytates and tannings contents by 73% and 88%, respectively, while the rate of reduction in phytates and tannins in microwave heating was ~84%. These results are comparable with the present study, wherein the findings elucidated significant decline in phytates, 86.2–11.6 mg/100 g, and tannins, 1175–170.0 mg/100 g, respectively, on processing the raw cabbage with microwave heating. This apparent decline in anti-nutrients could be attributed to the greater extent of water solubility and thermal degradability nutrients–toxicants complexes. Leafy vegetables, if addressed adequately for their intrinsic nutrient inhibition factors such as oxalates, may act as sustainable, highly acceptable and economical sources of micronutrients for the poor and malnourished populations of the developing countries. Food-based approaches to combat hidden hunger and other forms of malnutrition are more acceptable with regard to their cultural acceptability. Microwave-treated cabbage leaves and the powder, as had been developed in this study, can therefore find their place in regular cuisines as savory ingredients, micronutrient dense sprinkles, and as a bulking agent for low fiber meals.

Weaker legislative control over sanitary and phytosanitary practices for the field crops in Pakistan results in injudicious application of insecticides. Such practices eventually result in higher magnitude of multiple pesticides in plant foods. A higher rate of accumulation of pesticides residues in different parts of the food plants such as leaves, vascular fluids, grains and seeds could be ascribed to the natural translaminar and xylem-phloem movements of poisonous pesticides [34]. Furthermore, higher availability of the listed pesticides within the plant vascular tissues could also be attributed to their higher water solubility, higher surface adsorption and xylem transport features. The levels of pesticides identified in non-treated or raw cabbage powder clearly violates the MRLs identified by Codex and the EU and may be considered a matter of consumer safety concern. Microwave heat processing and other processing operations anticipated remarkable role in bringing down the MRL of imidacloprid, cypermethrin, bifenthrin and chlorpyrifos in cabbage to a safer limit. However, none of the listed processing technique was found satisfactory in limiting deltamethrin MRL to a safe consumption level. Prior to our study, comparable findings were reported by Athulya et al. [12], wherein washing and boiling as processing techniques reduced diafenthiuron residues in cabbage by 67% and 100%, respectively. Randhawa et al. [24] led a study from Pakistan, which presented slightly lower rates of reduction in pesticide residues of cauliflower by washing and cooking, i.e., 28% and 51%, respectively. In line with the above-mentioned findings of the recent study, earlier reports [35] had also attributed hydrolytic rate constants, water solubility, volatility, water–octanol and partition coefficient, dissolution ability, thermo-degradable nature, strength of chemical solution and vascular–translaminar movement of the pesticides to significantly affect rate of reduction in the pesticides residues in plant materials.

Value additions of the baked goods with novel ingredients are usually expected to significantly influence sensory attributes and consumer acceptability of the finished products. Reduction in taste of cabbage powder supplemented unleavened flatbread may be associated with isothiocyanates that anticipate sharp flavor and pungency to the consumer goods. Supplementation of CP at a concentration higher than 7.5% reduce taste acceptability score that may be linked to increased concentration of astringency factors including glucosinolates, isothiocyanates and tannins. Likewise, increasing CP supplementation level beyond 7.5% in flatbreads may also increase levels of hydrophilic groups in CP–wheat flour composite dough, thus improving the texture of the baked goods. Cabbage powder supplementation (5%) to wheat flour for white bread production and incorporating CP in beef patties (6%) have been previously recommended as acceptable by consumers groups for all sensory traits [5]. As has been referred to earlier, cabbage, like other vegetables of the cruciferous family, is a good carrier of fibers, phenolics and secondary plant metabolites such as glucosinolates, which can yield significant changes to color, taste and textural aspects of the processed products.

## 5. Conclusions

The key findings of this study suggest CP is likely to be a good source of minerals, dietary fiber, and plant protein that can anticipate significant health ameliorative properties when served alone or in combination. However, the presence of higher concentrations of anti-nutrients and pesticide residues beyond permissible limits in leafy green vegetables, resulting in nutritional disparities and high rate of acute to chronic health disorders. Present investigation depicted all the processing treatments to mitigate anti-nutrients and pesticides residues in raw CP and resultantly improved microelements contents of the processed powdered cabbage. The findings of the study are suggestive of microwave heating to exhibit promising outcomes in not merely reducing the burden of intrinsic toxins but also a major fraction of the pesticide residues. The results further conclude the application of microwave-processed CP as a natural food fortificant and a sustainable, culturally acceptable and economical means to mitigate micronutrients disparities and public health disorders including obesity.

## Figures and Tables

**Table 1:** Functional properties of raw and processed cabbage powder.

Treatments	BD (g/mL)	RR	WSI (%)	WAI (g/g)	Sp (g/g)	Hg (%)
Raw	0.69 ± 0.01 ^c^	4.79 ± 0.04 ^a^	5.10 ± 0.10 ^a^	6.81 ± 0.04 ^b^	7.74 ± 0.01 ^d^	7.56 ± 0.07 ^b^
Microwave Heating	0.67 ± 0.11 ^c^	4.76 ± 0.00 ^a^	4.02 ± 0.02 ^e^	6.91 ± 0.01 ^a^	8.00 ± 0.05 ^b^	7.60 ± 0.00 ^a^
Blanching	0.71 ± 0.02 ^b^	4.05 ± 0.05 ^c^	4.52 ± 0.02 ^c^	6.34 ± 0.00 ^c^	8.21 ± 0.02 ^a^	7.00 ± 0.50 ^c^
Acid Soaking	0.72 ± 0.01 ^ab^	4.28 ± 0.05 ^b^	4.21 ± 0.02 ^d^	6.31 ± 0.01 ^c^	7.85 ± 0.01 ^c^	6.89 ± 0.01 ^d^
Alkali Soaking	0.73 ± 0.03 ^a^	4.12 ± 0.03 ^c^	4.82 ± 0.04 ^b^	6.00 ± 0.00 ^d^	7.05 ± 0.05 ^e^	6.50 ± 0.10 ^e^

Values are means ± S.E. (n = 2). Values bearing identical lettering in each column are non-significant (*p* < 0.05), BD = Bulk density, RR = Rehydration ratio, WSI = Water solubility index, WAI = Water absorption index, Sp = Swelling power, Hg = Hygroscopicity.

**Table 2:** Proximate composition of raw and processed cabbage powder (g/100 g).

Treatments	Moisture	Ash	Protein	Fat	Fiber	Carbohydrates	Caloric Values (kcal/100 g)
Raw	10.64 ± 0.13 ^a^	9.44 ± 0.51 ^b^	12.18 ± 0.19 ^a^	1.72 ± 0.05 ^a^	25.18 ± 0.19 ^a^	40.83 ± 0.04 ^d^	227.5 ± 1.00 ^c^
Microwave Heating	10.05 ± 0.05 ^b^	10.35 ± 0.04 ^ab^	11.05 ± 0.05 ^b^	1.16 ± 0.05 ^b^	24.10 ± 0.10 ^b^	43.28 ± 0.19 ^c^	227.8 ± 0.97 ^c^
Blanching	9.55 ± 0.05 ^c^	10.25 ± 0.25 ^ab^	12.05 ± 0.05 ^a^	1.05 ± 0.07 ^b^	23.21 ± 0.02 ^c^	43.88 ± 0.20 ^b^	233.2 ± 0.41 ^b^
Acid Soaking	9.05 ± 0.06 ^d^	10.80 ± 0.20 ^a^	10.10 ± 0.10 ^c^	0.83 ± 0.03 ^c^	22.00 ± 0.00 ^e^	47.22 ± 0.02 ^a^	236.8 ± 0.75 ^a^
Alkali Soaking	8.84 ± 0.14 ^d^	10.66 ± 0.21 ^a^	9.99 ± 0.01 ^c^	1.19 ± 0.01 ^b^	22.58 ± 0.02 ^d^	46.74 ± 0.11 ^a^	236.6 ± 0.31 ^a^

Values are means ± S.E. (n = 2). Values bearing identical lettering in each column are non-significant (*p* < 0.05).

**Table 3:** Mineral composition of raw and processed cabbage powder (mg/100 g).

Treatments	Na	Ca	K	Fe	Zn
Raw	51.8 ± 0.29 ^a^	355 ± 0.50 ^a^	286 ± 1.00 ^ab^	13.7 ± 0.11 ^a^	32.0 ± 0.00 ^a^
Microwave heating	49.9 ± 0.34 ^ab^	347 ± 2.00 ^b^	279 ± 0.20 ^b^	13.4 ± 0.20 ^ab^	30.4 ± 0.15 ^ab^
Blanching	50.6 ± 0.40 ^ab^	339 ± 1.00 ^c^	279 ± 1.22 ^b^	13.0 ± 0.10 ^bc^	31.8 ± 0.15 ^a^
Acid soaking	50.1 ± 0.38 ^ab^	351 ± 1.00 ^ab^	272 ± 0.50 ^c^	13.1 ± 0.10 ^c^	31.6 ± 0.98 ^a^
Alkali soaking	48.9 ± 1.12 ^b^	348 ± 2.50 ^b^	266 ± 1.00 ^d^	13.6 ± 0.01 ^ab^	29.2 ± 0.45 ^b^

Values are means ± S.E. (n = 2). Values bearing identical lettering in each column are non-significant (*p* > 0.05).

**Table 4:** Anti-nutritional factors in raw and processed cabbage powders (mg/100 g).

Treatments	Alkaloids	Oxalates	Tannins	Phytates
Raw	184 ± 2.05 ^a^	48.9 ± 1.69 ^a^	1175 ± 2.06 ^a^	86.2 ± 1.92 ^a^
Microwave heating % Reduction	41.0 ± 1.00 ^d^ 77	7.32 ± 0.56 ^e^ 85	170.0 ± 3.0 ^d^ 85	11.6 ± 0.27 ^e^ 86
Blanching % Reduction	59.0 ± 1.01 ^c^ 68	15.2 ± 0.50 ^d^ 69	246 ± 6.0 ^c^ 79	15.8 ± 0.82 ^d^ 82
Acid soaking % Reduction	59.0 ± 1.05 ^c^ 68	18.6 ± 0.56 ^c^ 62	274 ± 2.50 ^b^ 76	19.6 ± 0.27 ^c^ 77
Alkali soaking % Reduction	79.7 ± 0.31 ^b^ 56	27.6 ± 0.45 ^b^ 44	280 ± 5.50 ^b^ 76	24.6 ± 0.81 ^b^ 71

Values are means ± S.E. (n = 2). Values bearing identical lettering in each column are non-significant (*p* > 0.05). % reduction is the reduction compared to the raw CP. % reduction represents the rate of reduction in antinutrients from the raw to the treated cabbage vis. microwave heated, blanched, acid or alkali soaked.

**Table 5:** Pesticide residues in raw and processed cabbage powders (ppm).

Treatments	Imidacloprid	Cypermethrin	Bifenthrin	Chlorpyrifos	Deltamethrin
Raw	0.98 ± 0.01 ^a^	1.22 ± 0.02 ^a^	1.03 ± 0.02 ^a^	1.97 ± 0.02 ^a^	2.12 ± 0.03 ^a^
Microwave heating % Reduction	0.12 ± 0.02 ^e^ 87	0.23 ± 0.01 ^e^ 80	0.15 ± 0.03 ^e^ 84	0.43 ± 0.03 ^e^ 77	0.36 ± 0.03 ^e^ 83
Blanching % Reduction	0.23 ± 0.01 ^d^ 75	0.34 ± 0.05 ^d^ 72	0.24 ± 0.01 ^d^ 74	0.61 ± 0.03 ^d^ 69	0.71 ± 0.04 ^d^ 66
Acid soaking % Reduction	0.30 ± 0.06 ^c^ 69	0.61 ± 0.01 ^c^ 48	0.31 ± 0.03 ^c^ 71	0.79 ± 0.01 ^c^ 60	0.81 ± 0.01 ^c^ 62
Alkali soaking % Reduction	0.52 ± 0.02 ^b^ 47	0.68 ± 0.01 ^b^ 42	0.45 ± 0.02 ^b^ 57	0.91 ± 0.01 ^b^ 54	1.39 ± 0.02 ^b^ 33
MRL (mg/Kg)	0.5	1.0	0.4	1.0	0.1

Values are means ± S.E. (n = 2). Values bearing identical lettering in each column are non-significant (*p* > 0.05). % reduction represents the rate of reduction in pesticides residues from the raw to the treated cabbage vis. microwave heated, blanched, acid or alkali soaked. MRL—maximum residue limit [8,9].

**Table 6:** Organoleptic properties of microwave-treated cabbage powder supplemented flatbread.

Treatments	Appearance	Color	Taste	Texture	Folding Ability	Overall Acceptability
T_0_	8.09 ± 0.09 ^a^	7.74 ± 0.29 ^a^	7.90 ± 0.22 ^a^	7.76 ± 0.21 ^a^	8.10 ± 0.09 ^a^	7.98 ± 0.15 ^a^
T_1_	6.95 ± 0.88 ^c^	6.92 ± 0.46 ^b^	6.62 ± 0.59 ^b^	6.81 ± 0.56 ^b^	6.69 ± 0.84 ^b^	6.80 ± 0.34 ^c^
T_2_	7.59 ± 0.32 ^b^	7.51 ± 0.19 ^a^	7.52 ± 0.33 ^a^	7.53 ± 0.24 ^a^	7.65 ± 0.42 ^a^	7.56 ± 0.28 ^b^
T_3_	6.06 ± 0.46 ^d^	5.87 ± 0.28 ^c^	6.01 ± 0.45 ^c^	6.10 ± 0.30 ^cd^	6.18 ± 0.32 ^c^	6.04 ± 0.26 ^d^
T_4_	5.95 ± 0.51 ^d^	5.41 ± 0.55 ^d^	6.04 ± 0.44 ^c^	5.67 ± 0.48 ^d^	5.74 ± 0.48 ^c^	5.76 ± 0.31 ^d^

Values are means ± S.D. (n = 20). Values bearing identical lettering in each column are non-significant (*p* < 0.05); 20 panelists tested the product. T_0_ = 100% whole wheat flour flatbread (control), T_1_ = 2.5% cabbage powder (CP), T_2_ = 5% CP, T_3_ = 7.5% CP, T_4_ = 10% CP.

## Data Availability

The date are available from the corresponding author.

## References

[1] Samec D., Pavlović I., Salopek-Sondi B. (2017). White cabbage (*Brassica oleracea* var. *capitata* f. *alba*): Botanical, phytochemical and pharmacological overview. Phytochem. Rev..

[2] Novotny C., Schulzova V., Krmela A., Hajslova J., Svobodova K.M., Koudela M. (2018). Ascorbic acid and glucosinolate levels in new Czech cabbage cultivars: Effect of production system and fungal infection. Molecules.

[3] Liang J.L., Yeow C., Teo K.C., Gnanaraj C., Chang Y.P. (2019). Valorizing cabbage (*Brassica oleracea* L. var. *capitata*) and capsicum (*Capsicum annuum* L.) wastes: In vitro health-promoting activities. J. Food Sci. Technol..

[4] Xu F., Zheng Y., Yang Z., Cao S., Shao X., Wang H. (2014). Domestic cooking methods affect the nutritional quality of red cabbage. Food Chem..

[5] Lee S.H. (2010). Effect of cabbage powder on baking properties of white breads. Korean J. Food Preserv..

[6] Rokayya S., Li C., Zhao Y., Li Y., Sun C. (2013). Cabbage (*Brassica oleracea* L. var. *capitata*) Phytochemicals with antioxidant and anti-inflammatory potential. Asian Pac. J. Cancer Prev..

[7] Amir R.M., Randhawa M.A., Nadeem M., Ahmed A., Ahmad A., Khan M.R., Kausar R. (2019). Assessing and reporting household chemicals as a novel tool to mitigate pesticide residues in spinach (*Spinacia oleracea*). Sci. Rep..

[8] Codex Alimentarius Commission (2011). International Food Standards. https://www.fao.org/fao-who-codexalimentarius/codex-texts/dbs/pestres/commodities-detail/en/?c_id=275.

[9] EU Commission Regulations (2011). Commission Regulation (EU) No. 524/2011. https://eur-lex.europa.eu/legal-content/EN/ALL/?uri=CELEX%3A32011R0524.

[10] Wanwimolruk S., Duangsuwan W., Phopin K., Boonpangrak S. (2017). Food safety in Thailand 5: The effect of washing pesticide residues found in cabbages and tomatoes. J. Consum. Prot. Food Saf..

[11] Samtiya M., Aluko R.E., Dhewa T. (2020). Plant food anti-nutritional factors and their reduction strategies: An overview. Food Prod. Process. Nutr..

[12] Athulya R., Kang B.K., Sahoo S. (2019). Effect of washing and boiling on pesticide residue reduction of diafenthiuron in Brinjal and cabbage. J. Entomol. Zool. Stud..

[13] Singh S., Swain S., Singh D.R., Salim K.M., Nayak D., Roy S.D. (2015). Changes in phytochemicals, anti-nutrients and antioxidant activity in leafy vegetables by microwave boiling with normal and 5% NaCl solution. Food Chem..

[14] Amin K., Akhtar S., Ismail T. (2018). Nutritional and organoleptic evaluation of functional bread prepared from raw and processed defatted mango kernel flour. J. Food Process. Preserv..

[15] Shafi M., Baba W.N., Masoodi F.A., Bazaz R. (2016). Wheat-water chestnut flour blends: Effect of baking on antioxidant properties of cookies. J. Food Sci. Technol..

[16] Yu L., Ramaswamy H.S., Boye J. (2013). Protein rich extruded products prepared from soy protein isolate-corn flour blends. LWT Food Sci. Technol..

[17] Sharma P., Gujral H.S., Rosell C.M. (2011). Effects of roasting on barley β-glucan, thermal, textural and pasting properties. J. Cereal Sci..

[18] American Association of Cereal Chemists (AACC) (2000). Approved Methods of the American Association of Cereal Chemists.

[19] Gani A., Wani S.M., Masoodi F.A., Salim R. (2013). Characterization of rice starches extracted from Indian cultivars. Food Sci. Technol. Int..

[20] Cai Y.Z., Corke H. (2000). Production and properties of spray-dried Amaranthus betacyanin pigments. J. Food Sci..

[21] Latimer J. (2019). Official Methods of Analysis.

[22] Onwuka G.I. (2006). Soaking, boiling and antinutritional factors in pigeon peas (*Cajanus cajan*) and cowpeas (*Vigna unguiculata*). J. Food Process. Preserv..

[23] Haug W., Lantzsch H.J. (1983). Sensitive method for the rapid determination of phytate in cereals and cereal products. J. Sci. Food Agric..

[24] Randhawa M.A., Anjum F.M., Asi M.R., Butt M.S., Ahmed A., Randhawa M.S. (2007). Removal of endosulfan residues from vegetables by household processing. J. Sci. Ind. Res..

[25] Chandra S., Singh S., Kumari D. (2015). Evaluation of functional properties of composite flours and sensorial attributes of composite flour biscuits. J. Food Sci. Technol..

[26] David O., Arthur E., Kwadwo S.O., Badu E., Sakyi P. (2015). Proximate composition and some functional properties of soft wheat flour. Int. J. Innov. Res. Sci. Eng. Technol..

[27] Prokopov T., Goranova Z., Baeva M., Slavov A., Galanakis C.M. (2015). Effects of powder from white cabbage outer leaves on sponge cake quality. Int. Agrophys..

[28] Warman P.R., Havard K.A. (1997). Yield, vitamin and mineral contents of organically and conventionally grown carrots and cabbage. Agric. Ecosyst. Environ..

[29] Umoh E.O., Iwe M.O. (2014). Effects of processing on the nutrient composition of false yam (*Icacina trichantha*) flour. Niger. Food J..

[30] Kimura M., Itokawa Y. (1990). Cooking losses of minerals in foods and its nutritional significance. J. Nutr. Sci. Vitaminol..

[31] Mosha T.C., Gaga H.E., Pace R.D., Laswal H.S., Mtebe K. (1995). Effect of blanching on the content of anti-nutritional factors in selected vegetables. Plant Foods Hum. Nutr..

[32] Zhong Y., Wang Z., Zhao Y. (2015). Impact of radio frequency, microwaving, and high hydrostatic pressure at elevated temperature on the nutritional and antinutritional components in black soybeans. J. Food Sci..

[33] Suhag R., Dhiman A., Deswal G., Thakur D., Sharanagat V.S., Kumar K., Kumar V. (2021). Microwave processing: A way to reduce the anti-nutritional factors (ANFs) in food grains. LWT.

[34] Nasr H.M., Abbassy M.A., Marzouk M.A., Mansy A.S. (2014). Determination of imidacloprid and tetraconazol residues in cucumber fruits. J. Pollut. Eff. Control.

[35] Akhtar S. (2013). Food Safety Challenges—A Pakistan’s Perspective. Crit. Rev. Food Sci. Nutr..

